# Risk of childlessness in help-seeking men with Peyronie’s disease—A Swedish longitudinal study

**DOI:** 10.1371/journal.pone.0315948

**Published:** 2025-01-30

**Authors:** Ralf Kuja-Halkola, Lars Henningsohn, Brendan Zietsch, Henrik Larsson, Martin Cederlöf

**Affiliations:** 1 Department of Medical Epidemiology and Biostatistics (Solna), Karolinska Institutet, Stockholm, Sweden; 2 Department of Clinical Science, Intervention, and Technology (CLINTEC), Karolinska Institutet, Stockholm, Sweden; 3 School of Psychology, University of Queensland, Brisbane, Australia; 4 School of Medical Sciences, Faculty of Medicine and Health, Örebro University, Örebro, Sweden; Dicle University, TÜRKIYE

## Abstract

Peyronie’s disease (PD) is a disorder of the penis that is associated with poor mental health, lowered psychosocial- and sexual wellbeing, which may increase the risk of childlessness in men affected by the disorder. Although this is an issue of significant clinical importance, it has not been addressed in research to date. We conducted a longitudinal cohort study based on data from Swedish national registers utilizing a large sample of help-seeking men with PD, along with matched subjects from the general population. We assessed the probability and odds ratio of childlessness, modeled with logistic regressions, and offspring rate ratio, modeled with Poisson regression. We found that the probability of childlessness was somewhat lowered for men with PD aged between 35 and 71 years at end of follow-up. Men with PD aged 35 or less showed slightly elevated probabilities of childlessness. Specifically, odds ratios for childlessness were between 0.5 and 1.0 for men aged above 35, and between 1 and 1.5 for men aged less than 35, although the confidence intervals for increased odds partly included the null. Analyses of men’s rate of offspring showed similar pattern, with higher rate ratios for older men and lower for younger men. Although more research is needed, the findings of this study suggest that clinical urological practice may be enhanced by a proactive discussions about the potential issue of childlessness in younger men with PD.

## Introduction

Peyronie’s disease (PD) is characterized by the formation of fibrous tissue, “plaques”, in the tunica albuginea of the corpora cavernosa in the penis, resulting in a bent, shortened or abnormally shaped erection that may impair intercourse [1,2]. Despite long-standing research, the etiology of the disorder remains obscure but may be influenced by genetics [3], pro-inflammatory processes [4], and immunologic factors [5]. The typical of onset of the disorder occurs around the age of 50 years [6], but the variability is substantial and even teenagers may be affected [7]. The prevalence of PD is uncertain and may approach 9% [8], although 3.2% was the estimate reported in a large, widely cited questionnaire study [9]. Ample evidence suggests a major influence of PD on mental health [10–12], psychosocial well-being [10,12], and sexual well-being [13,14], so relationship strain and difficulties might be expected [5]. Consequently, PD may also affect reproduction. This possibility is reinforced by findings in a previous population-based study from our group: we found excess risks of several diagnosed psychiatric disorders that are associated with reduced offspring rate [11]. The relationship between mental health problems and sexual functioning in men with PD was examined in a qualitative study by Rosen and colleagues [12]. They found empirical evidence of a conceptual model where the physical appearance of the penile deformity has negative effects on self-esteem, sexual desire and function, and interpersonal relationships, and that lowered self-esteem further influenced sexual desire and function, as well as interpersonal relationship functioning [12]. To our best knowledge, the issue of increased risk of childlessness in PD has not been addressed in previous research. This knowledge gap was the rationale for the study, and the objective was to conduct a longitudinal cohort study to assess the risk of childlessness in a large sample of help-seeking Swedish men with PD, by utilizing data from Swedish national registers.

## Materials and methods

In accordance with Swedish law and the Swedish Ethics Review Authority, the requirement for informed consent was waived as this study was based on data from national registers. Following ethical approval from the Regional Ethics Committee in Stockholm, Sweden, we conducted a longitudinal cohort study with PD diagnoses as the exposure and risk of childlessness as the outcome, using the Swedish male population. All methods were performed in accordance with the relevant guidelines and regulations. We modelled: 1) risk of childlessness, and 2) offspring rate.

### Study cohort

We performed a register linkage, using the unique personal identification numbers held by all Swedish residents. From the Total Population Register [15] we identified all men born 1942–1991 who were living in Sweden in January 1997, when PD diagnoses became available in the Swedish diagnostic system, N = 2,881,512. The men could have immigrated prior to 1997, but not emigrated ever before 1997. We observed date of birth for all offspring of these men, both before and after 1997 – in total 4,087,840 children. The men were thus. We followed the men until emigration, death, or end of follow-up (December 31^st^ 2013), whichever occurred first. Thus, we included all men who we could follow from the time of PD diagnosis being observable and did not exclude men until the date when they were censored from our dataset – no exclusions were made based on other characteristics of the men. End of follow-up was limited by our data access, but the chosen birth years allowed us to observe a range of ages of men at end of follow-up, making it possible to investigate patterns of childlessness and offspring rate over differentially aged men.

### Study variables and national registers

Using the unique personal identity number, we linked the cohort to the National Patient Register (NPR) [16], where we identified PD diagnosis using the tenth version of the International Classification of Diseases (ICD-10; implemented in Sweden in 1997), code N48.6. All diagnoses in the NPR are assigned by the treating medical doctor, either at discharge from an inpatient care episode (data accessible since 1997), or at a visit at a specialist outpatient unit (data accessible since 2001), and followed the diagnostic criteria in the ICD-10. The diagnoses of chronic disorders in the NPR are generally valid, with positive predictive values between 85% and 95% [17]. Although the PD diagnoses have not been specifically validated, the disorder is clinically characteristic and easily diagnosed by the presence of palpable plaques which rule out potential differential diagnoses. Hence, the validity of the PD diagnoses in the NPE are likely valid measurements. We observed birth date and migrations from the Total Population Register and deaths from the Cause of Death Register [15,17]. We used the Multi-Generation Register [17] to identify all offspring born to the men studied. The Multi-Generation Register has excellent coverage for identifying fathers, covering 94% of fathers of individuals born in 1947, and 98% in 1961 and with increasing proportions with increasing calendar time. Furthermore, misattributed paternity in Sweden has been estimated at only 1.7%, decreasing with calendar time to 1% in children born in 2010 or later [16]. For the purposes of computing offspring rate, we counted multiple births (twins, triplets, and higher) as one childbirth event, but when adjusting for number of previous children as covariate we allowed the count of children to increase according to the number of children born. This was done since we were more interested in whether men were able to conceive children than how many children they produced.

### Descriptive statistics

We summarized data by ever being diagnosed with PD. We categorized the men’s birth years in 10-year intervals and summarized the absolute number and percent of men in each category. We counted total number of offspring per man, and summarized total number and proportion with 0, 1, 2, 3, 4, 5, and ≥6 children. We also identified those men who were childless, i.e., those with 0 children, at end of follow-up (December 31^st^ 2013) and calculated the absolute number and percentage by birth year categories. Finally, we calculated the mean and standard deviation of the number of children per man, by birth year categories.

### Statistical analyses

#### Childlessness.

We fitted models of childlessness at the end of follow-up using logistic regression. In these models, PD diagnosis was treated as a binary never/ever exposure, regardless of at what age the men had received the diagnosis. The reason for treating PD diagnosis as a binary exposure was to avoid being overly reliant on PD diagnosis date; where identification of a diagnosis in specialist care may happen long after disease onset. Moreover, the limitation of not being able to observe diagnoses before 1997 would likely lead to many men having the disease before start of follow-up, and the observable diagnosis date would represent a date not relating to disease onset, and this would be differentially problematic depending on men’s age (more problematic for older men, less so for younger men). The age at end of follow-up varied considerably and ranged from 22 years (those born in 1991) to 71 years (those born in 1942), wherefore the probabilities of childlessness varied substantially over birth years. We modelled the probability of childlessness by exposure status as odds ratios, which were allowed to vary flexibly over time, using a natural cubic spline (with 6 degrees of freedom and knots placed according to evenly spaced percentiles of birth year), and adjusted for a main effect of birth years (again, using natural cubic splines with 6 degrees of freedom). We also calculated the probability of childlessness by PD status from these models.

#### Offspring rate.

We modelled the offspring rate using Poisson regression. Starting from January 1997, we split the age of the participants through follow-up into intervals; <20 years, five-year intervals up until 60, and ≥60. To model the offspring rate, i.e., number of offspring per person-years, we used number of childbirths (i.e., we only counted twins, triplets, and higher as one childbirth) per age interval as outcome, and time in age interval as offset term, meaning that the offspring rate of men with PD was compared with men without PD for each age interval. We utilized the longitudinal nature of the data and treated PD diagnosis as time-varying, so that time before diagnosis was considered unexposed and time after diagnosis as exposed, and we ended follow-up at death, emigration, or end of study period. We then modelled the association between PD and childbearing rate flexibly over birth year, using natural cubic splines (with 6 degrees of freedom). We adjusted each time interval for number of previous children (categorized as above), and a main effect of birth year, using natural cubic splines (with 6 degrees of freedom).

#### Sensitivity analyses.

Since some individuals emigrated during follow-up, we re-performed the childlessness analyses using only those who did not emigrate before end of follow-up (still including those who died, since their final number of children was known).

We performed two additional sensitivity analyses using time-to-event analysis (i.e., survival analysis). In these analyses we assessed the relative “risk” of having a child as hazard ratios using Cox regression. We treated the exposure, PD diagnosis, as time-varying; men were considered unexposed before (a potential) PD diagnosis and exposed after (they remained unexposed through follow-up if they did not have a PD diagnosis). We started follow-up January 1^st^, 1997, when exposure to PD diagnosis could be measured. First, we analyzed **time to first child** among men childless January 1^st^, 1997. For this analysis, because of lacking statistical power, we had to limit the birth years to those born 1957–1991. Second, we analyzed the **time to next child** for all offspring born after start of follow-up to all men (i.e., each childbirth was counted as a new outcome event). In this analysis we adjusted for number of previous children. For both analyses we modelled the hazard ratio flexible over birth years using natural cubic splines (with 5 degrees of freedom).

In all analyses, we accounted for deviations from distributional assumptions in the models and dependencies between rows of data (e.g., several children from one man, and that men were brothers) using cluster robust standard errors, where the clusters were the ID of the men’s mothers (if no mother was identifiable, we created a unique cluster for that man).

## Results

### Descriptive statistics

Descriptive statistics are shown in Table 1. A total of 6,809 help-seeking men with PD were identified, along with 2,874,703 men without PD. The proportion of childlessness in men with PD ranged from 11.2% to 85.2% across birth cohorts, and from 19.3% to 82.6% in men without PD. Childlessness was less common in men with PD compared with men without PD in all birth cohorts, except for the latest born cohort (men born 1982–1991). The mean number of offspring in men with PD ranged from 2.13 (SD 1.24) to 0.20 (SD 0.57) across birth cohorts, and 1.87 (SD 1.32) to 0.25 (SD 0.61) in men without PD. The mean number of offspring was higher in men with PD than in men without PD in all birth cohorts, with the exception of members of the youngest category.

**Table 1:** Numbers and proportions of birth cohort members, childlessness, offspring, and mean (standard deviation) of number of offspring, by PD.

	Peyronie’s disease N (%)	Not Peyronie’s disease N (%)
Peyronie’s disease	6,809 (0.2)	2,874,703 (99.8)
Birth year [age at end of follow up]		
1942–1951 [62–71]	2,803 (41.2)	623,790 (21.7)
1952–1961 [52–61]	1,909 (28.0)	571,328 (19.9)
1962–1971 [42–51]	1,154 (16.9)	611,787 (21.3)
1972–1981 [32–41]	639 (9.4)	517,671 (18.0)
1982–1991 [22–31]	304 (4.5)	550,127 (19.1)
Childlessness* [age at end of follow up]		
1942–1951 [62–71]	313 (11.2)	120,625 (19.3)
1952–1961 [52–61]	278 (14.6)	121,499 (21.3)
1962–1971 [42–51]	200 (17.3)	142,415 (23.3)
1972–1981 [32–41]	214 (33.5)	185,655 (35.9)
1982–1991 [22–31]	259 (85.2)	454,598 (82.6)
Number of offspring		
0	1,264 (18.6)	1,024,792 (35.6)
1	1,024 (15.0)	417,424 (14.5)
2	2,497 (36.7)	881,340 (30.7)
3	1,367 (20.1)	389,064 (13.5)
4	445 (6.5)	112,292 (3.9)
5	137 (2.0)	32,601 (1.1)
≥6	75 (1.1)	17,190 (0.6)
Mean (standard deviation) number of offspring [age at end of follow up]		
1942–1951 [62–71]	2.13 (1.24)	1.87 (1.32)
1952–1961 [52–61]	2.14 (1.40)	1.88 (1.37)
1962–1971 [42–51]	1.87 (1.24)	1.72 (1.27)
1972–1981 [32–41]	1.25 (1.11)	1.24 (1.14)
1982–1991 [22–31]	0.20 (0.57)	0.25 (0.61)

Note: Men were alive and in Sweden in January 1997.

*Percent of those born the specific years.

#### Childlessness.

**Fig 1:**
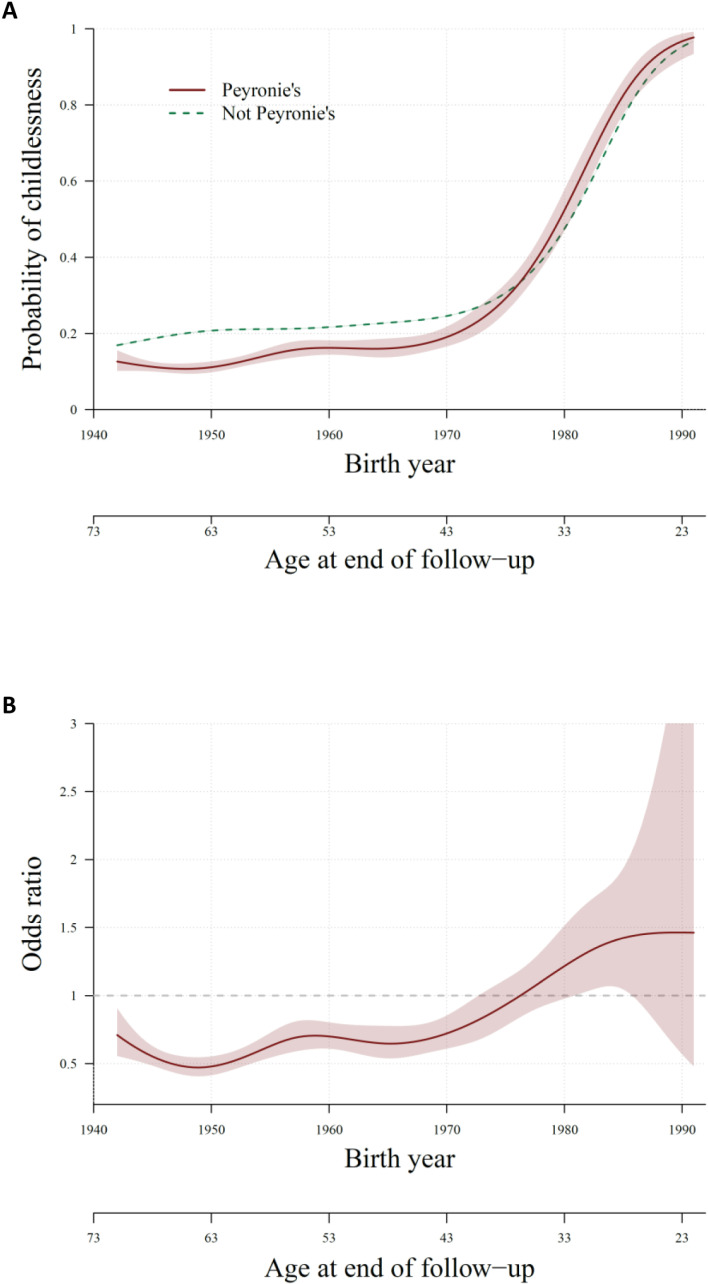
Probability of childlessness at end of follow-up (panel A) and odds ratio of childlessness (panel B) for men with versus without Peyronie’s disease, by birth year and age at end of follow-up. Estimate and 95% confidence interval.

Fig 1, Panel **A** shows the modelled probabilities of being childless at end of follow-up by birth year, and age at end of follow-up, and Fig 1, Panel **B** shows the odds ratios based on these probabilities.

The probability of childlessness was lower for men with PD born 1942 to approximately 1978, aged approximately 35 to 71 years at end of follow-up, compared with men without PD. For men born approximately 1978 and onwards, the probability of childlessness in men with PD was higher than for men without PD, while probability in both groups greatly increased to above 80% in men born in around 1984–1985 or later, aged about 28 or younger at end of follow-up (Panel **A**). Hence, the odds ratios of childlessness for men with PD, compared with men without PD, varied between 0.5 and 1.0 for men born 1942 to approximately 1978, with the confidence interval mostly not including the null. The odds ratio varied between 1.0 and 1.5 for those born after 1978 (aged below 35 at end of follow-up), but with wide confidence intervals that included the null for most birth years (Panel **B**).

#### Offspring rate.

**Fig 2:**
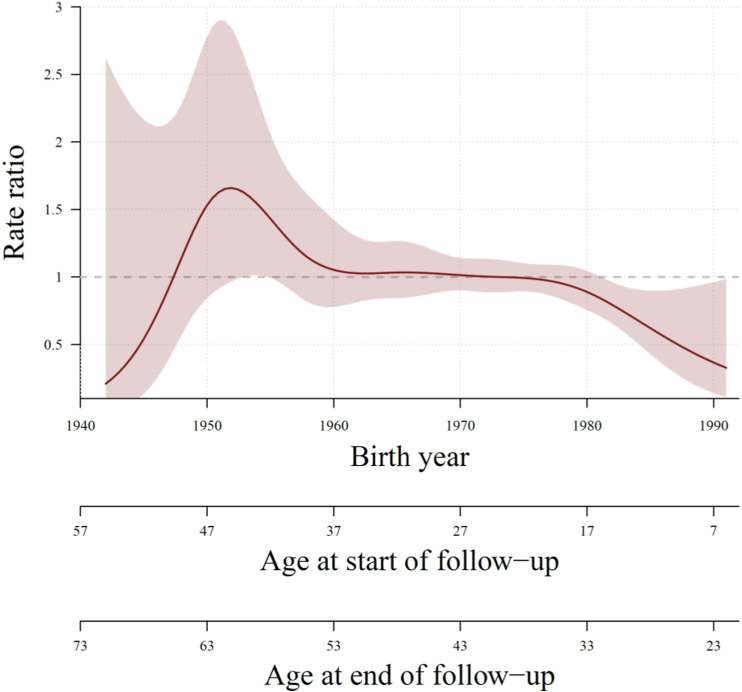
Offspring rate ratios for men with versus without Peyronie’s disease. Estimate and 95% confidence interval.

Fig 2 shows the offspring rate ratios for men with PD compared with comparison subjects.

For men born 1942, aged 55 years at the start of follow-up and 71 years at the end of follow-up, the offspring rate ratio was far below 1, indicating fewer offspring, but not statistically significant with a wide confidence interval up to about 2.5. The offspring rate ratio increased to over 1.5 for men born around 1950, i.e., men with PD had about 50% increased rate of offspring compared to men without PD, with a confidence interval of about 1 to 3. For men born about 1960 to 1978, the rate ratio curve flattened out and approximated 1, and for men born 1978 to 1990 and aged about 7–19 years at the start of follow-up, and 23–35 years at end of follow-up, the offspring rate ratio decreased to below 1, indicating that younger men with PD had fewer children, statistically significant with a confidence interval not including 1.

### Sensitivity analyses

Results of the analyses of participants with complete follow-up are presented in S1 Fig. The curves of the probability of childlessness at follow-up (Panel **A**), as well as the curve of the odds ratio of childlessness (Panel **B**) at the end of follow-up remained similar to the curves in the main analyses. Results for time-to-event analysis for time to first child is found in S2 Fig. For time to first child the analysis supported the higher childlessness in relatively younger men aged less than 35 at end of follow-up (i.e., the hazard ratio was lower than 1 indicating that men with PD had fewer children), but not among the youngest men aged 25 or less at end of follow-up. The lower proportion of childless men among older men was not seen, probably due to restriction to childless men at start of follow-up. Results for time-to-event analysis for time to next child, using all children born to the included men, is found in S3 Fig. Compared to the rate of offspring analysis we observed the same lower rate of children among younger men, but for oldest men, aged above 50 at end of follow-up, we observed a slightly increased risk of additional offspring.

## Discussion

In this longitudinal cohort study comprising 2.8 million Swedish men and their more than 4 million children, we found only marginal differences at the end of follow-up with respect to the probability of childlessness, the odds ratio of childlessness, as well as the offspring rate ratio for men with PD, compared with men without PD. In other words, the risk of childlessness in help-seeking men diagnosed with PD in inpatient or specialist outpatient care did not seem to be elevated, with the exception of younger men (aged less than 35). To our best knowledge, the current study is the first investigation of this issue.

In our study, the men with PD had been diagnosed in specialist care settings, presumably most commonly at outpatient urology clinics. What are the characteristics of this cohort? First, the participants are help seeking, which means that they are likely to be sexually active, and to have identified their disorder as a problem. For some, the problem may be of a sexual nature with difficulties related to anything from lowered sexual confidence to painful erections. Others may experience a degree of penile curvature that makes vaginal intercourse painful, awkward, difficult, or even impossible, and therefore directly affect the fertility of these men. In any case, our results show than men with PD who end up in the care of urologist do not seem to differ from men without PD in terms with our outcome measurements.

It is important to note that our data does not allow for inferences of the treatment success, e.g., surgery aimed at reducing penile curvature that may underlie the observed results. Although, the slight exception seen in our data was that younger men with PD diagnosis (aged about 35 or younger at end of follow-up) had lower offspring rate and larger probability of being childless. One possible explanation of this finding is that young men with PD who are in stable relationships and in the process of starting a family and get (biological) children are more likely to seek help for their PD compared to similarly aged men with PD who are not in stable relationships and/or trying to conceive a child (i.e., that the lower rates of children represent a selection effect into those who get their PD diagnosis early). It is possible, based on the higher rate of children in older men, that these men will eventually be able produce offspring to the extent they would like, either through surgery or other interventions, i.e., they may “catch up” at a slightly higher age. Another possible explanation is that young men with PD may be less likely to engage in serious relationships due to self-stigmatization. PD is an embarrassing condition that often has negative effects on the self-esteem and self-confidence of affected men, not only related to sexuality, but in a much broader, self-defining, existential sense. In qualitative research, men with PD have described the disorder as “freakish”, and descriptions of their self-image include words like being “like a cripple”, “half a man”, and “abnormal” [10]. This means that some men with PD are likely to avoid contacts with health care due to self-stigmatization, and that PD is likely to be under-diagnosed in the general population. For similar reasons, these men with PD, who we did not capture as being diagnosed with PD in our data set, may also avoid sexual relationships and thus be far more likely to be childless due to their condition. This possibility will be important to address, ideally by large-scale questionnaire studies probing PD symptomatology and relevant measurements of childlessness in nationally representative samples. Furthermore, qualitative research on the topic, ideally targeting men with PD as well as their potential partners, are imperative. Taken together, while more research is needed, but the findings from this study highlights the need for practitioners in primary care, as well as in specialist care, to ask their PD patients about problems related to intimate relationships and reproduction and assess the need for referral to relationship counseling.

This study has several strengths; it is based on unique data from several Swedish national registers, well known for their validity and usefulness in research. Furthermore, the data is longitudinal, and the sample size is particularly large. This study also has several limitations that should be considered when interpreting the results and discussion; we lacked follow-up through the full fertile period for a substantial proportion of the cohort, and our data did not allow for inferences of the risk of childlessness of men with PD who did not present any contacts with specialist care. Further, the differential coverage of ages when observing the PD diagnosis, depending on birth year and time when diagnosis was observable, necessarily hampers the comparison across different ages of follow-up and diagnosis, leading to potential differences in disease presentation, severity, and etiology. Nevertheless, the results remain valid for each age, even if one need to interpret differences between ages with caution. Additionally, the timing of observed diagnosis may be sub-optimal with regards to capturing disease onset; in the main analysis of risk of childlessness we disregarded time to circumvent being too reliant on the diagnosis date, which may have led to other problems regarding timing of diagnosis compared to timing of birth of children. We performed sensitivity analyses taking the time of diagnosis into account using survival analysis with time-varying exposure to address this – the results remained largely comparable to the main analyses. Finally, the PD diagnoses have not been formally validated, which leaves a concern about its validity.

## Conclusions

We observed that the risk of childlessness of help-seeking Swedish men with PD did not differ more than marginally from the comparison subjects without PD, with the notable exception of men with an early PD diagnosis, who showed an excess risk of childlessness. Large-scale questionnaire studies of the risk of childlessness of men with PD who tend to avoid contacts with health care are needed. Although more research is needed, the findings of this study suggest that clinical urologist should ask young men with PD about the potential issue of childlessness.

## Supporting information

S1 Fig Probability (panel A) and odds ratio (panel B) of childlessness for men with PD versus men without PD. Only men that did not emigrate or die during follow-up are included, 5,998 men with PD and 2,516,107 without. Estimate and 95% confidence interval.
(DOCX)

S2 Fig Survival analysis of time to first child among men who were childless at start of follow-up (January 1st 1997). Hazard ratio with 95% confidence intervals.
(DOCX)

S3 Fig Survival analysis of time to next child for all children born after start of follow-up (January 1st 1997) among all men. Analysis adjusted for number of prior children. Hazard ratio with 95% confidence interval.
(DOCX)

## References

[1] Sasso F, Gulino G, Falabella R, D’Addessi A, Sacco E, D’Onofrio A, et al. Peyronie’s disease: lights and shadows. Urol Int. 2007;78(1):1–9. doi: 10.1159/000096927 17192725

[2] Ziegelmann MJ, Bajic P, Levine LA. Peyronie’s disease: contemporary evaluation and management. Int J Urol. 2020;27(6):504–16. doi: 10.1111/iju.14230 32253786

[3] Sharma KL, Alom M, Trost L. The etiology of Peyronie’s disease: pathogenesis and genetic contributions. Sex Med Rev. 2020;8(2):314–23. doi: 10.1016/j.sxmr.2019.06.004 31540807

[4] Byström J, Rubio C. Induratio penis plastica Peyronie’s disease. Clinical features and etiology. Scand J Urol Nephrol. 1976;10(1):12–20. doi: 10.3109/00365597609179648 1273527

[5] Schiavino D, Sasso F, Nucera E, Alcini E, Gulino G, Milani A, et al. Immunologic findings in Peyronie’s disease: a controlled study. Urology. 1997;50(5):764–8. doi: 10.1016/S0090-4295(97)00333-6 9372889

[6] Mulhall JP, Schiff J, Guhring P. An analysis of the natural history of Peyronie’s disease. J Urol. 2006;175(6):2115–8; discussion 2118. doi: 10.1016/S0022-5347(06)00270-9 16697815

[7] Tal R, Hall MS, Alex B, Choi J, Mulhall JP. Peyronie’s disease in teenagers. J Sex Med. 2012;9(1):302–8. doi: 10.1111/j.1743-6109.2011.02502.x 21981606

[8] Mulhall JP, Creech SD, Boorjian SA, Ghaly S, Kim ED, Moty A, et al. Subjective and objective analysis of the prevalence of Peyronie’s disease in a population of men presenting for prostate cancer screening. J Urol. 2004;171(6 Pt 1):2350–3. doi: 10.1097/01.ju.0000127744.18878.f1 15126819

[9] Schwarzer U, Sommer F, Klotz T, Braun M, Reifenrath B, Engelmann U. The prevalence of Peyronie’s disease: results of a large survey. BJU Int. 2001;88(7):727–30. doi: 10.1046/j.1464-4096.2001.02436.x 11890244

[10] Nelson CJ, Mulhall JP. Psychological impact of Peyronie’s disease: a review. J Sex Med. 2013;10(3):653–60. doi: 10.1111/j.1743-6109.2012.02999.x 23153101

[11] Kuja-Halkola R, Henningsohn L, D’Onofrio BM, Mills J, Adolfsson A, Larsson H, et al. Mental disorders in Peyronie’s disease: a Swedish cohort study of 3.5 million men. J Urol. 2021;205(3):864–70. doi: 10.1097/JU.0000000000001426 33081594

[12] Rosen R, Catania J, Lue T, Althof S, Henne J, Hellstrom W, et al. Impact of Peyronie’s disease on sexual and psychosocial functioning: qualitative findings in patients and controls. J Sex Med. 2008;5(8):1977–84. doi: 10.1111/j.1743-6109.2008.00883.x 18564146

[13] Levine LA. The clinical and psychosocial impact of Peyronie’s disease. Am J Manag Care. 2013;19(4 Suppl):S55–61. 23544796

[14] Weidner W, Schroeder-Printzen I, Weiske WH, Vosshenrich R. Sexual dysfunction in Peyronie’s disease: an analysis of 222 patients without previous local plaque therapy. J Urol. 1997;157(1):325–8. doi: 10.1016/s0022-5347(01)65370-9 8976290

[15] Ludvigsson JF, Almqvist C, Bonamy A-KE, Ljung R, Michaëlsson K, Neovius M, et al. Registers of the Swedish total population and their use in medical research. Eur J Epidemiol. 2016;31(2):125–36. doi: 10.1007/s10654-016-0117-y 26769609

[16] Ludvigsson JF, Andersson E, Ekbom A, Feychting M, Kim J-L, Reuterwall C, et al. External review and validation of the Swedish national inpatient register. BMC Public Health. 2011;11:450. doi: 10.1186/1471-2458-11-450 21658213 PMC3142234

[17] Ekbom A. The Swedish multi-generation register. Methods Mol Biol. 2011;675:215–20. doi: 10.1007/978-1-59745-423-0_10 20949391

