# Supplementary material for: Risk of childlessness in help-seeking men with Peyronie’s disease—A Swedish longitudinal study
Source: PLoS One. 2025 Jan 30;20(1):e0315948. doi: 10.1371/journal.pone.0315948 (PMC11781693; doi:10.1371/journal.pone.0315948)
Supplement: S1 Fig — Only men that did not emigrate or die during follow-up are included, 5,998 men with PD and 2,516,107 without. Estimate and 95% confidence interval. (DOCX) [file pone.0315948.s001.docx]

Supplementary information

Risk of childlessness in help-seeking men with Peyronie’s disease – a Swedish longitudinal study

Ralf Kuja-Halkola^1^,PhD, Lars Henningsohn^2^, MD, PhD, Brendan Zietsch^3^ , PhD, Henrik Larsson^4,1^, PhD, Martin Cederlöf, ^4,1^, PhD.

^1^ Department of Medical epidemiology and Biostatistics (Solna), Karolinska Institutet, Stockholm, Sweden.

^2^ Department of Clinical Science, Intervention, and Technology (CLINTEC), Karolinska Institutet, Stockholm, Sweden

^3^ School of Psychology, University of Queensland, Brisbane, Australia

^4^ School of Medical Sciences, Faculty of Medicine and Health, Örebro University, Örebro, Sweden.

Corresponding author: Martin Cederlöf, PhD.

**S1Fig.** Probability (panel **A**) and odds ratio (panel **B**) of childlessness for men with PD versus men without PD. Only men that did not emigrate or die during follow-up are included, 5,998 men with PD and 2,516,107 without. Estimate and 95% confidence interval.

| **A**   |
| --- |
| **B**   |
