# Supplementary material for: Risk of childlessness in help-seeking men with Peyronie’s disease—A Swedish longitudinal study
Source: PLoS One. 2025 Jan 30;20(1):e0315948. doi: 10.1371/journal.pone.0315948 (PMC11781693; doi:10.1371/journal.pone.0315948)
Supplement: S2 Fig — Hazard ratio with 95% confidence intervals. (DOCX) [file pone.0315948.s002.docx]

**SFig2.** Survival analysis of time to first child among men who were childless at start of follow-up (January 1^st^ 1997). Hazard ratio with 95% confidence intervals.
