# Supplementary material for: Risk of childlessness in help-seeking men with Peyronie’s disease—A Swedish longitudinal study
Source: PLoS One. 2025 Jan 30;20(1):e0315948. doi: 10.1371/journal.pone.0315948 (PMC11781693; doi:10.1371/journal.pone.0315948)
Supplement: S3 Fig — Analysis adjusted for number of prior children. Hazard ratio with 95% confidence interval. (DOCX) [file pone.0315948.s003.docx]

**SFig3.** Survival analysis of time to next child for all children born after start of follow-up (January 1^st^ 1997) among all men. Analysis adjusted for number of prior children. Hazard ratio with 95% confidence interval.
